# Effect of Non-Thermal Atmospheric Pressure Plasma (NTP) and Zirconia Primer Treatment on Shear Bond Strength between Y-TZP and Resin Cement

**DOI:** 10.3390/ma13183934

**Published:** 2020-09-05

**Authors:** Jong-Ju Ahn, Dae-Sung Kim, Eun-Bin Bae, Gyoo-Cheon Kim, Chang-Mo Jeong, Jung-Bo Huh, So-Hyoun Lee

**Affiliations:** 1 Department of Prosthodontics, Dental Research Institute, Dental and Life Science Institute, Pusan National University, Yangsan 50612, Korea; tarov0414@hanmail.net (J.-J.A.); modesthanks@gmail.com (D.-S.K.); 0228dmqls@hanmail.net (E.-B.B.); cmjeong@pusan.ac.kr (C.-M.J.); neoplasia96@hanmail.net (J.-B.H.); 2 Department of Oral Anatomy and Cell Biology, School of Dentistry, Pusan National University, Yangsan 50612, Korea; ki91000m@pusan.ac.kr; 3 Research & Development Center, FEAGLE Corporation, Yangsan 50614, Korea

**Keywords:** non-thermal atmospheric pressure plasma (NTP), yttria-stabilized tetragonal zirconia polycrystal, surface treatment, zirconia primer, sandblasting, dentistry, bonding

## Abstract

The purpose of this study was to investigate the effect of non-thermal atmospheric pressure plasma (NTP) treatment on the sandblasting of mechanical method and zirconia primer of chemical method used to increase the bond strength between zirconia and resin cement. In this study, Y-TZP was divided into 4 groups according to the surface treatment methods as follows: Zirconia primer (Pr), NTP + Zirconia primer (NTP + Pr), Sandblasting + Zirconia primer (Sb + Pr), Sandblasting + NTP + Zirconia primer (Sb + NTP + Pr). Then, two types of resin cement (G-CEM LinkAce and Rely X-U200) were used to measure the shear bond strength (SBS) and they were divided into non-thermal cycling group and thermal cycling group for aging effect. Statistical analyses were performed using the Kruskal-Wallis test and Mann-Whitney U test. The result of the surface energy (SE), there was no significant difference among the groups (*p* > 0.05). As a result of the SBS test, the Sb + Pr group had a significantly higher SBS value than the other groups regardless of the resin cement type (*p* < 0.05), and the decrease rate after thermal cycling treatment was the lowest. On the other hand, the NTP + Pr group showed significantly lower SBS values than the other groups except for the case of using Rely X-U200 (*p* < 0.05), and the reduction rate after thermal cycling was the highest. The Sb + NTP + Pr group did not differ significantly from the Pr group (*p* > 0.05). Within the limitations of two successive studies, treatment with NTP after sandblasting used for mechanical bond strength showed a positive effect on initial SBS. However, when NTP was treated before the zirconia primer used for the chemical bond strength, it showed a negative effect on SBS compared to other treatment methods, which was noticeable after the thermal cycling treatment.

## 1. Introduction

As the CAD-CAM system was developed and patients’ interest in aesthetics was being increased, Yttria-stabilized tetragonal zirconia polycrystal (Y-TZP) was being commonly used in ceramic core material because its favorable physical and esthetic properties [1,2,3]. The feldspathic ceramic restorations, which was commonly used before Y-TZP was developed, obtained mechanical retention using hydrofluoric acid etching and chemically altered the surface using primer containing silane coupling agent to increase bond strength with a resin cement [4,5]. However, these methods were not suitable for application to silica-free Y-TZP [6,7]. Therefore, there was a need to develop a method of increasing the bond strength between Y-TZP and resin cement [8].

Hydrochloric acid containing ferric ion (Fe^3+^) or the copper ion (Cu^2+^) can locally corrode the Y-TZP surface, creating microporosity on the surface, increasing adhesion [9]. However, it is dangerous for the experimenter to handle these corrosive substances directly, and acid etching of the restoration has a negative effect between cement and Y-TZP [10,11]. Sandblasting, a common mechanical method used to increase Y-TZP bond strength [12,13,14], uses alumina air abrasion to increase the roughness of the Y-TZP surface and provide resin cement and micromechanical interlocking [15,16]. However, this method can damage the inner surface of Y-TZP, negatively affecting long-term success [15]. The use of zirconia primer containing 10-methacryloyloxydecyl dihydrogen phosphate (10-MDP) has been introduced as another chemical method to increase the mechanism of Y-TZP adhesion [7,17]. Although this primers have been reported to increase adhesion by chemical interactions between the Y-TZP surface and resin cement [18,19], some studies have shown that the bond strength of sintered Y-TZP surface-treated with zirconia primers is significantly reduced after aging process [20,21]. Therefore, no consensus yet exists for what is the effective bonding method to Y-TZP prosthesis [22,23,24].

Recently, non-thermal plasma (NTP) treatment has attracted in the dental field, such as dental material sterilization and surface modification [25,26]. Surface modification of materials using plasma has been used in various industries for decades [27]. NTP is an ionized gas composed of electrons, free radicals, and ions [21]. It is known that when applied to materials, the structure of the material surface can be chemically modified by functional groups produced by chemical reactions without compromising the mechanical properties of the surface [4,28,29,30,31]. It has been found that treatment with NTP can increase the surface energy (SE) of zirconia and promote chemical interaction, thereby increasing the bonding strength with resin cement [32,33]. In our previous study, it was confirmed that the surface contact angle decreased and the surface energy increased when Y-TZP was treated with NTP, but NTP alone treatment did not show a significant effect on improving the bonding strength with resin cement [34]. It can also be confirmed that it is more effective to increase the bonding strength of Y-TZP and resin cement when using both sandblasting and NTP than sandblasting alone [34]. However, previous studies could only confirm the effect of the mechanical method and the interaction of NTP on the bond strength. There have been few studies on the effect of NTP on the treatment of zirconia primers used as chemical methods.

Therefore, the purpose of this study was to investigate the effect of NTP treatment on the mechanical and chemical methods used to increase the bond strength between zirconia and resin cement. After the sandblasting and zirconia primers and NTP were combined on the surface of Y-TZP, the degree of surface energy change of the treated Y-TZP was measured and the shear bond strength between Y-TZP and resin cement was evaluated.

## 2. Materials and Methods

### 2.1. Preparation of Specimens

**Figure 1:**
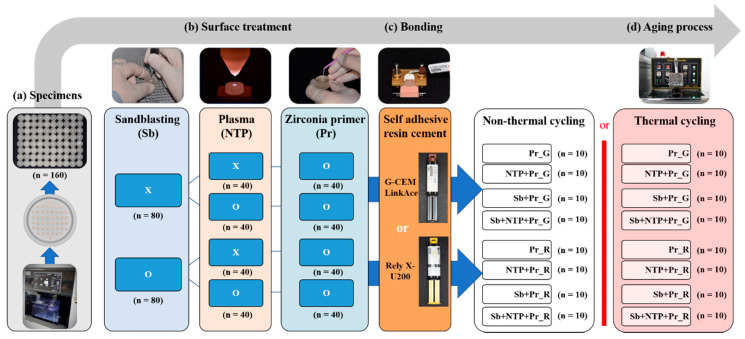
Specimens processing method and distribution flow for shear bond strength (SBS) experiment. (**a**) Fabrication of the disc type specimens (**b**) Surface treatment methods; (**c**) Bonding of Y-TZP surface and resin cement; (**d**) Thermal cycling process for aging.

The 24 fully sintered Y-TZP cubes (10 mm length, 10 mm width, and 10 mm height) (LUXEN, DentalMax, Seoul, Korea) were prepared to analyze the surface energy and 160 fully sintered Y-TZP discs (5 mm diameter, 3 mm height) were prepared for shear bond strength. For all disc type specimens, all surfaces except for the top were embedded into an acrylic resin (Orthodontic resin, Dentsply, Konstanz, Germany) to be treated (Figure 1a). All specimens were polished to 600 grit and 800 grit silicon carbide paper under water cooling using a grinding machine (MetaServ 250, Buehler, Lake Blu, IL, USA).

### 2.2. Surface Treatment

Then Y-TZP cubes and discs were randomly distributed in 4 experimental groups according to surface treatment as follow:

- Group Pr: Y-TZP surface + Zirconia primer
- Group NTP + Pr: Y-TZP surface + NTP treatment + Zirconia primer
- Group Sb + Pr: Y-TZP surface + Sandblasting + Zirconia primer
- Group Sb + NTP + Pr: Y-TZP surface + Sandblasting + NTP treatment + Zirconia primer

**Figure 2:**
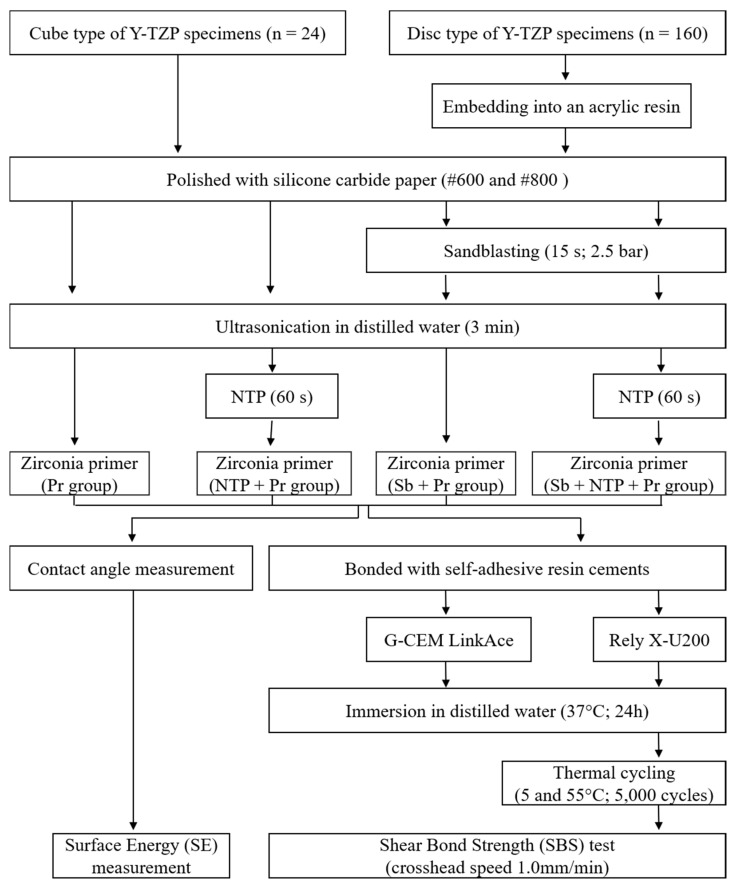
Flowchart of the study design.

The sandblasting was performed with dental sandblaster (Basic master, Renfert, Hilzingen, Germany) using 50 µm aluminum oxide (Al_2_O_3_) (Hi-Aluminas, Shofu Inc., Kyoto, Japan) at 2.5 bar for 15 s (Figure 1b) [34]. After sandblasting, all the Y-TZP specimens were ultrasonically cleaned in distilled water for 3 min and air-dried. NTP was supplied to specimens using a plasma generating device (FG-Explorer, FEAGLE, Yangsan, Korea) with argon (Ar) gas, which was operated at a flow rate of 5.0 slm (standard liter per minute), 3 kVpp, and 4.80 mA. Additionally, NTP treatment was implemented 10 mm above of the specimen surface for 1 min (Figure 1b). Then, Zirconia primer (Z-Prime Plus, Bisco Inc., Schaumberg, IL, USA), containing 10-Methacryloyloxydecyl dihydrogen phosphate (10-MDP), was applied to surface of specimens using microbrush and air-dried with an air syringe for 5 s (Figure 1b). Figure 2 summarizes the study protocol, and Table 1 shows how the groups are divided.

### 2.3. Contact Angle and Surface Energy (SE) Measurements

**Figure 3:**
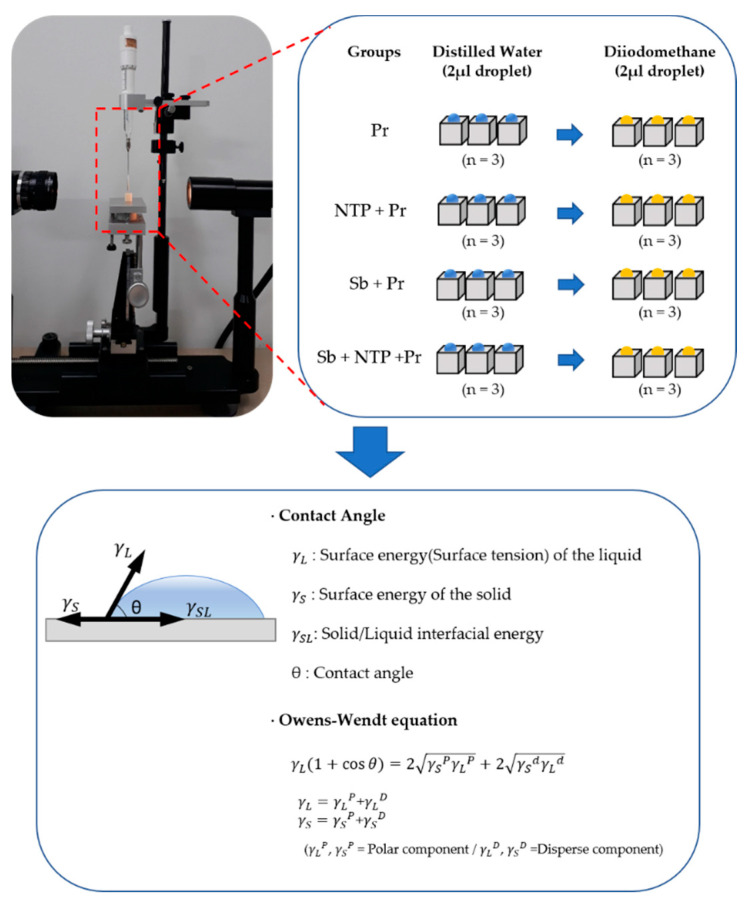
The process of measuring surface energy in each group.

Two different test liquids were used to measure the contact angle: deionized water (polar component), diiodomethane (dispersive component) (Tokyo Chemical Industry Co., Tokyo, Japan) [34]. The 24 Y-TZP cubes were divided into four groups according to surface treatment of the specimens (Pr group, NTP + Pr group, Sb + Pr group, Sb + NTP + Pr group) and immediately tested after the surface treatments. The contact angle was measured with computer-controlled image analyzer equipped with a video camera (Ramé-Hart 190-U1, Ramé-Hart Instrument Co., Succasunna, NJ, USA) by the sessile drop technique. The contact angle was measured three times for each specimen with two kinds of liquids, respectively, and average the values. At each measurement, the Y-TZP cubes were dried with absorbent paper. The surface energy of each specimen was calculated by the Owens-Wendt method, which calculated the contact angle by, $r_{s}=r_{s}^{p}+r_{s}^{d}$, being $r_{s}$ the combination SE of the polar component and dispersive component, being $r_{s}^{p}$ the polar component of the SE, being $r_{s}^{d}$ the dispersive component of the SE. The protocol for measuring the surface energy in this study is shown in Figure 3.

### 2.4. Shear Bond Strength (SBS) Test

#### 2.4.1. Bonding Y-TZP with Self-Adhesive Resin Cement

**Figure 4:**
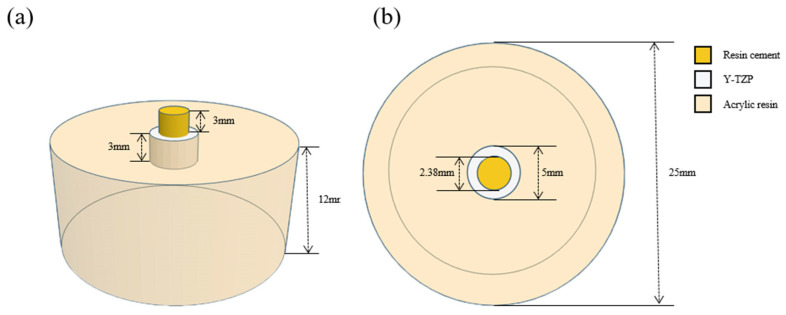
Schematic figure of the shear bond test specimen. (**a**) Frontal view of the specimen; (**b**) Top view of the specimen.

Two types of self-adhesive resin cements were used to perform SBS test: G-CEM LinkAce (GC Corporation, Tokyo, Japan) and Rely X-U200 (3M ESPE, St. Paul, MN, USA). The information of these cements is shown in Table 2. The 160 fully sintered Y-TZP discs were primarily classified into four groups: Pr group (*n* = 40), NTP + Pr group (*n* = 40), Sb + Pr group (*n* = 40) and Sb + NTP + Pr group (*n* = 40) according to the surface treatment method, and secondly divided into two sub-groups within the group (*n* = 20) depending on the type of resin cement used. The resin cements were bonded to the Y-TZP in a uniform size using an Ultradent jig (Ultradent Products Inc., South Jordan, UT, USA) with a 2.38 mm diameter and a 3 mm height (Figure 4). Subsequently, the resin cement was light-cured (Elipar™ DeepCure-L, 3M ESPE, St. Paul, MN, USA) for 20 s at 1000–1200 mW/cm^2^. Then, all specimens were immersed in 37 °C distilled water for 24 h.

#### 2.4.2. Aging Process and SBS Test

The group, first divided by surface treatment method (*n* = 40) and secondly by the type of resin cement used (*n* = 20), is thirdly divided into two sub-groups (*n* = 10), depending on whether or not each group is subjected to thermal cycling. While the non-thermal cycling group was tested immediately after storage, the Thermal cycling group was subjected to 5000 thermal cycling processes (5–55 °C, 30 s dwell time per bath). The SBS test (ISO 29022:2013) was performed using shear bond test (Bisco Inc., Schaumburg, IL, USA) at a 1.0 mm/min crosshead speed [34]. Shear bond strengths (MPa) were calculated by dividing the maximum force that induced failure by the bonded area.

### 2.5. Failure Mode Analysis

After SBS test, the surfaces of specimens were observed using an optical microscope (BX51, Olympus, Tokyo, Japan) at ×100 magnification. Failure modes were classified as an adhesive failure occurring at resin cement to Y-TZP interface, cohesive failure occurring in resin cement, and mix failure through a combination of these failures. The representative failure specimens according to each surface treatment method were evaluated by field-emission scanning electron microscope (Fe-SEM, Supra 25, Zeiss, Jena, Germany).

### 2.6. Statistical Analysis

To analyze the effects of the surface treatment on the surface energy, Kruskal-Wallis analysis was used. The Mann Whitney test and Kruskal-Wallis analysis were conducted to analyze the SBS results value according to the cement type, thermal cycling, and surface treatment method. Statistical analysis was performed using statistical analysis software (ver. 25.0, IBM, Armonk, NY, USA) with a confidence level of 0.05.

## 3. Results

### 3.1. Surface Energy (SE) Analysis

**Figure 5:**
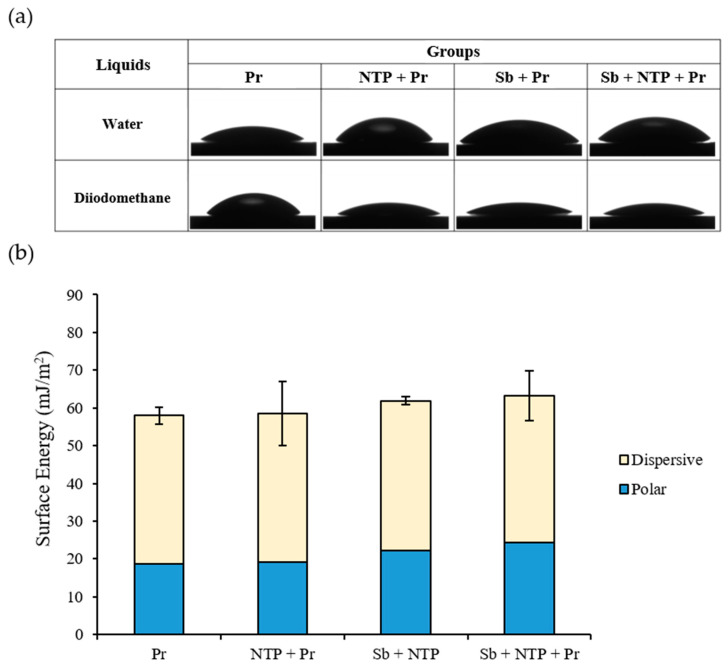
(**a**) Image of measuring contact angle of each group using water and diiodomethane. (**b**) Means of surface energy and its polar and dispersive part of each group. Pr, Zirconia primer; NTP + Pr, NTP + Zirconia primer; Sb + Pr, Sandblasting + Zirconia primer; Sb + NTP + Pr, Sandblasting + NTP + Zirconia primer.

Figure 5a shows a representative contact angle image of each group. The angle values of each group were similar. The means ± standard deviations (SD) of SE for Pr, NTP + Pr, Sb + Pr, and Sb + NTP + Pr were 57.98 ± 2.19, 61.87 ± 1.10, 58.56 ± 8.48, 63.21 ± 6.57, respectively (*p* > 0.05) (Table 3) (Figure 5b).

### 3.2. Shear Bond Strength (SBS) Analysis

**Figure 6:**
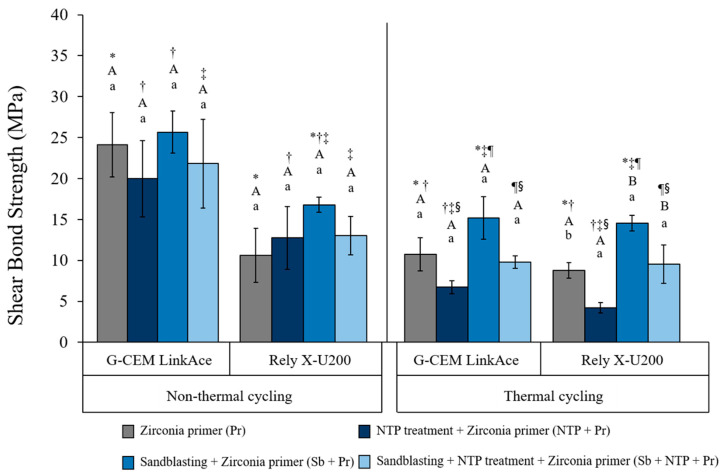
The results of SBS for all experimental groups according to surface treatment methods, effect of thermal cycling and the type of self-adhesive resin cement. The same symbols (*,†, ‡, ¶, §) indicate a significant difference between surface treatment within the same cement type of the same thermal effect (*p* < 0.05). The same upper case letters (A, B) indicate a significant difference between cement types within the same surface treatment of the same thermal effect (*p* < 0.05). The same lower case letters (a) indicate a significant difference between thermal effect within the same surface treatment of the same cement types. (*p* < 0.05). Pr, Zirconia primer; NTP+Pr, NTP + Zirconia primer; Sb+Pr, Sandblasting + Zirconia primer; Sb+NTP+Pr, Sandblasting + NTP + Zirconia primer.

The results of the SBS test were shown in Table 4 and Figure 6. First, when comparing SBS results from each surface treatment on the same cement within the same thermal effect, Sb + Pr group showed the highest value as follows: When using G-CEM, 25.66 ± 2.55 for without thermal cycling and 15.03 ± 2.56 for non-thermal cycling. When using Rely X-200, 16.62 ± 0.9 for without thermal cycling and 14.37 ± 0.93 for non-thermal cycling (*p* < 0.05). Second, when comparing the SBS values according to the type of cement used in the same surface treatment and same thermal effect, all groups of G-CEM LinkAce cement showed a significantly higher value than using Rely X-U200 cement (*p* < 0.05) (except the Sb + Pr group and the Sb + NTP + Pr group after thermal cycling). Third, the SBS results before and after thermal processing were compared within the same surface treatment for the same cement. The surface treatment method that changed the bonding strength of Y-TZP and resin cement the most after thermal cycling was NTP + Pr group, and the Sb + Pr group showed the least changed. In the case of G-CEM LinkAce cement, SBS decreased by 55.84% (Pr), 66.68% (NTP + Pr), 41.42% (Sb + Pr), and 55.45% (Sb + NTP + Pr) after heat treatment. For Rely X-U200 cement, SBS was reduced by 17.63% (Pr), 67.19% (NTP + Pr), 13.53% (Sb + Pr), 27.15% (Sb + NTP + Pr). From the reduction ratio, the G-CEM LinkAce group seems to have decreased more than Rely X-U200. However, compared to before the thermal cycling, it can be seen that G-CEM LinkAce group showed higher bonding strength than Rely X-U200 group (*p* < 0.05). Therefore, it seems that the ratio value of G-CEM LinkAce group was relatively lower after thermal cycling.

### 3.3. Failure Mode Analysis

**Figure 7:**
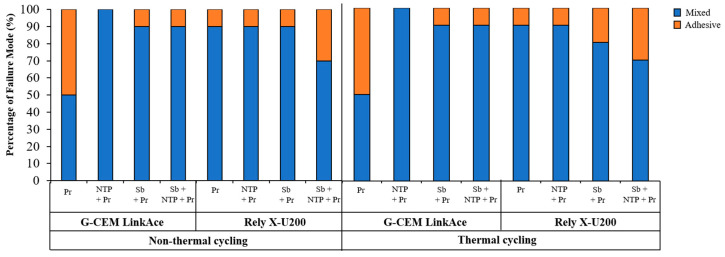
The percentage of failure mode of each group after SBS test. Pr, Zirconia primer; NTP + Pr, NTP + Zirconia primer; Sb + Pr, Sandblasting + Zirconia primer; Sb + NTP + Pr, Sandblasting + NTP + Zirconia primer.

**Figure 8:**
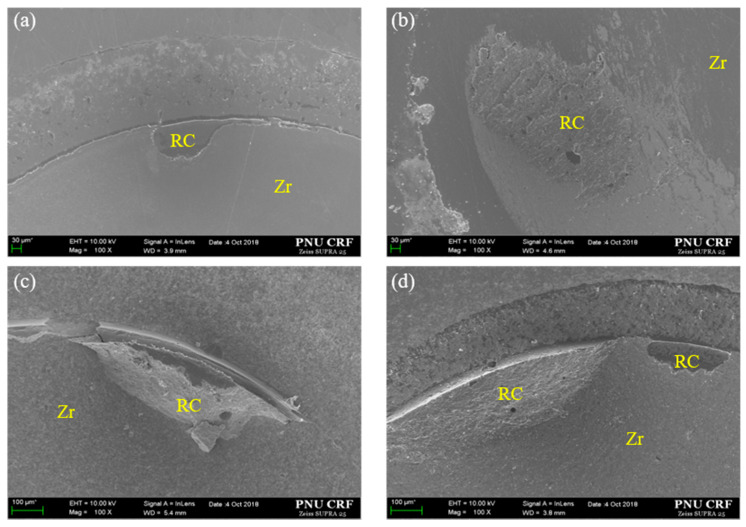
Representative FE-SEM images (×100) of the Y-TZP for each surface treatment method after thermal cycling and SBS test. (**a**) Pr, Zirconia primer; (**b**) NTP + Pr, NTP + Zirconia primer; (**c**) Sb + Pr, Sandblasting + Zirconia primer; (**d**) Sb + NTP + Pr, Sandblasting + NTP + Zirconia primer. (**a**–**d**) show mixed failures. RC, resin cement; Zr, Y-TZP surface.

The failure mode of all experimental groups is shown in Figure 7. An increase of adhesive failure mode after thermal cycling appeared at Sb + Pr group cemented with Rely X-U200 cement. In all other groups except this group, the percentage of adhesive and mixed type of failure mode was maintained after thermal cycling. Figure 8 shows representative images of each group that were classified according to treatment method. All images show the mixed type of failure mode.

## 4. Discussion

Y-TZP is a ceramic core that has excellent biocompatibility [35] and mechanical strength such as hardness [36] and wear resistance [37], so it is widely used in the dental field [6]. However, the use of Y-TZP is limited due to poor bonding strength with resin cement. In order to overcome this, the sandblasting method reported to increase the mechanical bond strength by removing contaminants and increasing the surface roughness [38] and the application method of zirconia primer, which is expected to induce chemical interaction and increase bonding strength, have been attempted [32]. Non-thermal atmospheric pressure plasma (NTP) treatment, which is known to increase the surface energy (SE) of a material and can sterilize the surface, is expected to increase the bonding strength between zirconia and resin cement, and many studies are being conducted [38,39]. The surface of material treated NTP is activated by energy that generated from the collisions using gas molecule with electrons, and gas molecules transform to ionized [40]. After an ionized gas molecule is exposed to air, it reacts with atmospheric oxygen or water, generating OH and O radicals [41]. Especially, argon gas used in this study has low metastable states [27,40], so it can form OH radicals at relatively low energy [42]. However, in the previous study, the SBS values were lower in the group treated with zirconia only with NTP compared to the group treated with zirconia only with sandblasting [34]. Therefore, the purpose of this study was to identify the interaction effects of sandblasting, zirconia primer, and NTP on the bonding strength between Y-TZP and resin cement using the previous experiment data (no treatment group, NTP group, sandblasting (Sb) group, and sandblasting with NTP (Sb+NTP) group) and the results of this study, which added primer surface treatment.

Plasma treatment has been reported to break C-C bonds and C-H bonds of zirconia surface, remove the contaminant, and increase oxygen atoms and decrease carbon atoms [39,43,44]. These changes in the surface properties can increase the hydrophilicity and thus improving the wettability of the primer or resin cement [45,46]. In our previous study, NTP treatment after sandblasting had the effect of increasing SE (Sb: 50.27, Sb + NTP: 71.47, *p* < 0.05) [34]. Therefore, in this study, NTP treatment was performed after sandblasting and before zirconia primer application. As a result of this study, there was no statistically significant difference in SE values of each group (*p* > 0.05), and the NTP treatment before primer application did not affect the change of SE (Pr: 57.98, NTP + Pr: 58.56, *p* > 0.05). This is similar to the results of another study [47]. All groups had the primers applied at the last step, so it is supposed that similar surface energy appeared for all groups.

To compare the bond strength through the oral environment simulation, the thermal cycling of 5000 cycles equal to approximately six months was performed [48]. According to the result of the SBS test before and after thermal cycling processing, it was observed that the SBS values of all groups were significantly lowered except for the Pr group using Rely X U-200 cement. (*p* < 0.05). Analyzing the degree of reduction after thermal cycling, in the case of G-CEM cement, SBS decreased as 55.84% (Pr), 66.68% (NTP + Pr), 41.42% (Sb + Pr) and 55.45% (Sb + NTP + Pr). For Rely X-U200 cement, SBS was reduced by 17.63% (Pr), 67.19% (NTP + Pr), 13.53% (Sb + Pr), 27.15% (Sb + NTP + Pr). It is supposed that the bonding strength of between zirconia and resin cement was not stable against the hydrolysis process in the thermal cycling [49]. In addition, since thermal cycling weakens the filler matrix binding strength according to the temperature change and brings out physical decomposition of the resin cement itself, it seems that the bonding strength decreases when thermal cycling [50].

With results according to the surface treatment method, it was confirmed that the combination of sandblasting and zirconia primer (Sb + Pr group, non-thermal cycling G-CEM LinkAce: 25.66 ± 2.55, Rely X-U200: 16.62 ± 0.9 and thermal cycling G-CEM LinkAce: 15.03 ± 2.56, Rely X-U200: 14.37 ± 0.93) is the most effective in both resin cement regardless of the thermal effect (*p* < 0.05). It is known that the treatment of Sb + Pr group increases roughness and surface area by sandblasting, and chemical modification is made by primer treatment, allowing resin cement to flow well through the microgap of Y-TZP surface and to enable chemical bonding by functional groups [15,16].

It was found that the additional NTP treatment (NTP + Pr group, Sb + NTP + Pr group) was not effective in improving the bonding strength with the resin cement. Regardless of the type of resin cement, these groups showed a significant decrease in SBS values after the thermal cycling process compared to the group without NTP treatment (Pr group, Sb + Pr group). It can be inferred that NTP treatment is not effective in such a case, and NTP may interfere with the proper reaction of Y-TZP and zirconia primer. In this experiment, a zirconia primer (Z-Prime Plus) containing 10-MDP to promote bond strength was used. The state of chemical bonding between 10-MDP and zirconia has not yet been fully determined, but the main chemical bonding states include covalent, ionic, metal, and chelation bonds [51]. Three models as the mechanisms of chemical interaction between zirconia primer containing 10-MDP and surface of Y-TZP was proposed [52]. Chemical bonding state of phosphate group of 10-MDP and zirconium oxide are as follows: (1) hydrogen bonding between the P=O (oxo group) of 10-MDP monomer and Zr–OH of zirconia surface (2) ionic bonding between the P–O^−^ group of deprotonated 10-MDP monomer and Zr^4+^ of zirconia and at the same time, intermolecular hydrogen bonding between the other P–OH group of 10-MDP and P=O group of another neighboring 10-MDP (3) ionic bonding between P–O^−^ group of 10-MDP and Zr^4+^ of zirconia and hydrogen-bonding interactions with Zr-OH via P=O (oxo group) of 10-MDP [53,54]. If NTP is pretreated, the zirconia surface is activated due to the formation of OH and O radicals, and the possibility of 10-MDP access may increase [45,46], but it can be considered that the chance of ionic or hydrogen bonding formed by direct reaction between the zirconia surface and the phosphoric acid group of the primer can be reduced. This potential mechanism does not show much effect in the initial reaction, but it can be inferred that hydrolysis occurred at a higher rate after the thermal cycling in which the oral environment was reproduced, and had a great effect on the SBS reduction effect.

Two types of self-adhesive resin cements were used in this study: G-CEM LinkAce and Rely X-U200, which are widely used in dental clinics these days. Tanış et al. [55] reported that the retention strength of the resin cements is determined by the type of phosphate monomer present in the cement composition. The manufacturer of G-CEM LinkAce announced that G-CEM LinkAce contains special ester phosphate monomers [56,57]. The manufacturer of Rely X-U200 stated that Rely X-U200 includes methacrylate monomers and phosphate groups [56,57]. The bonding ability of the phosphoric monomers is influenced by the length of the –(CH_2_)n chain of the monomer [57]. In the results of shear bond strength before thermal cycling, all groups using G-CEM LinkAce showed significantly higher values than those using Rely X-U200. The same results were obtained in our previous study. Based on the results of this experiment, it can be suggested that although the manufacturer did not reveal the specific components of each product, but different results were obtained due to the monomer characteristics of each product.

Similar to other previous studies [58,59], this research used shear bond strength to measure the bonding strength between resin cement and Y-TZP. We observed the failure mode to judge the bonding strength according to the failure mode type. Oyagüe et al. [58] have reported that mixed failure mode and cohesive failure mode mean having high bond strength value than adhesive failure mode. However, in our study, the failure mode pattern was similar despite the decrease in shear bond strength after thermal cycling in all groups. This might be that the shear bond strength test protocol can misjudge the occurring stress comparing with the clinical side and the irregular distribution of stresses within the adhesion region can lead to significant changes in failure mode [59].

According to previous studies, there is nothing clearly defined, but for the biomaterial to maintain masticatory force, it must have a shear bond strength of 5–50 MPa [60]. In the results of this study, except for the NTP + Pr group using Rely X-U200 with thermal cycling, the SBS values of all groups were included within 5–50 MPa, so it is supposed that there is no problem in maintaining mastication. However, there were limitations of this study, such as an insufficient number of specimens, a comparison of few surface treatment methods and resin cement types, and a lack of reproducibility in clinical situations. In this study, all the results were analyzed as non-parametric statistics. This is due to the insufficient number of specimens, and it is believed that further studies should proceed with the experiment with a larger number. In this study, self-adhesive resin cement was used for both resin cements, but the results were very different. This suggests that very different result values may be obtained depending on the difference in resin cement, so it is considered that the experiment should be conducted with more various resin cements in the future. In addition, although intraoral situation was reproduced in the laboratory through thermal cycling, it is supposed that the intraoral environment was not completely reproduced simply by changing the temperature and being immersed in water. Therefore, it is considered that verification through clinical trials is necessary to confirm whether it is effective in the oral cavity. It is thought that a standardized surface treatment method that can increase the bonding strength of Y-TZP and resin cement can be established through research conducted by supplementing these limitations.

## 5. Conclusions

Within the limitations of the present study, the combination of NTP and zirconia primer on SBS between zirconia and resin cement was less effective than when primer was used alone. As a result of our two consecutive studies, when NTP was treated after sandblasting used for mechanical bond strength, it showed a positive effect on initial SBS, but when NTP was treated before zirconia primer used for chemical bond strength, it showed a negative effect. Both before and after thermal cycling treatment, regardless of resin cement type, SBS of the combination of sandblasting and zirconia primer was the highest among all groups and reduction rate of thermal effect was the lowest. When sandblasting, NTP, and zirconia primers were used sequentially, the effect of each treatment method was canceled, and thus there was no significant difference from the use of primer alone.

## Figures and Tables

**Table 1:** Distribution of Y-TZP specimens for all groups.

Groups	Distribution of Y-TZP Specimens (n)				
	Surface Energy (SE)	Shear Bond Strength (SBS)			
		G-CEM LinkAce		Rely X-U200	
		Non-Thermal Cycling	Thermal Cycling	Non-Thermal Cycling	Thermal Cycling
Pr	3	10	10	10	10
NTP + Pr	3	10	10	10	10
Sb + Pr	3	10	10	10	10
Sb + NTP + Pr	3	10	10	10	10

Pr, Zirconia primer; Sb + Pr, Sandblasting + Zirconia primer; NTP + Pr, non-thermal atmospheric pressure plasma (NTP) + Zirconia primer; Sb + NTP + Pr, Sandblasting + NTP + Zirconia primer.

**Table 2:** Composition of used resin cement and primer in this study.

Material	Manufacturer	Type	Composition
G-CEM LinkAce	GC Corporation, Tokyo, Japan	Self-adhesive Dual-cure Automix	Paste A: Fluoroalumino silicate glass, Urethane dimethacrylate (UDMA), Dimethacrylate, Pigment, Silicon dioxide, Initiator, Inhibitor
			Paste B: Urethane dimethacrylate (UDMA), Dimethacrylate, Phosphoric acid ester monomer, Initiator, stabilizer
Rely X-U200	3M ESPE, St. Paul, MN, USA	Self-adhesive Dual-cure Automix	Base paste: Methacrylate monomers containing phosphoric acid groups, Methacrylate monomers, Silanated fillers, Initiator components, Stabilizers, Rheological additives
			Catalyst paste: Methacrylate monomers, Alkaline (basic) fillers, Silanated fillers, Initiator components, Stabilizers, Pigments, Rheological additives
Z-Prime Plus	Bisco Inc., Schaumberg, IL, USA	Zirconia primer	Ethanol, BisGMA, 2-Hydroxyethyl methacrylate, 10-Methacryloyloxydecyl dihydrogen phosphate (10-MDP)

**Table 3:** Minimum, median, maximum, and mean surface energy values (mJ/m^2^) and standard deviations (SD) for each group.

Groups	Minimum Surface Energy	Median Surface Energy	Maximum Surface Energy	Mean Surface Energy ± SD
Pr	55.53	58.64	59.76	57.98 ± 2.19
NTP + Pr	52.32	55.15	68.21	58.56 ± 8.48
Sb + Pr	60.65	62.16	62.79	61.87 ± 1.10
Sb + NTP + Pr	58.36	60.59	70.70	63.21 ± 6.57

**Table 4:** Minimum, median, maximum, and mean shear bond strength values (MPa) and standard deviations (SD) for each group.

Groups			SBS (Mpa)			
Thermal cycling	Cement type	Surface treatment	Minimum	Median	Maximum	Mean ± SD
Non Thermal cycling	G-CEM LinkAce	Pr	18.22	23.16	29.69	24.12 ± 3.95 *^,A,a^
		NTP + Pr	11.87	18.68	27.89	19.99 ± 4.67 ^†,A,a^
		Sb + Pr	21.51	25.98	28.90	25.66 ± 2.55 ^†,A,a^
		Sb + NTP + Pr	15.08	21.91	31.48	21.82 ± 5.43 ^‡,A,a^
	Rely X-U200	Pr	5.37	10.91	15.03	10.49 ± 3.26 *^,A,a^
		NTP + Pr	6.58	11.70	19.42	12.62 ± 3.82 ^†,A,a^
		Sb + Pr	15.44	16.57	18.43	16.62 ± 0.90 *^,†,‡,A,a^
		Sb + NTP + Pr	9.57	13.41	16.04	12.89 ± 2.34 ^‡,A,a^
Thermal cycling	G-CEM LinkAce	Pr	7.57	10.16	13.84	10.65 ± 2.01 *^,†,A,a^
		NTP + Pr	5.69	6.67	7.87	6.66 ± 0.81 ^†,‡,§,A,a^
		Sb + Pr	11.66	14.65	19.03	15.03 ± 2.56 *^,‡,¶,A,a^
		Sb + NTP + Pr	8.76	9.57	10.97	9.72 ± 0.75 ^¶,§,A,a^
	Rely X-U200	Pr	6.97	8.67	9.66	8.64 ± 0.94 *^,†,A,b^
		NTP + Pr	3.28	4.18	4.88	4.14 ± 0.61 ^†,‡,§,A,a^
		Sb + Pr	13.51	14.04	16.04	14.37 ± 0.93 *^,‡,¶,B,a^
		Sb + NTP + Pr	5.57	9.66	12.56	9.39 ± 2.33 ^¶,§,B,a^

The same symbols (*,†, ‡, ¶, §) indicate a significant difference between surface treatment within the same cement type of the same thermal effect (*p* < 0.05). The same upper case letters (A, B) indicate a significant difference between cement types within the same surface treatment of the same thermal effect (*p* < 0.05). The same lower case letters (a) indicate a significant difference between thermal effect within the same surface treatment of the same cement types. (*p* < 0.05). Pr, Zirconia primer; NTP+Pr, NTP + Zirconia primer; Sb + Pr, Sandblasting + Zirconia primer; Sb + NTP + Pr, Sandblasting + NTP + Zirconia primer.
